# Genome-wide methylation analysis reveals differentially methylated loci that are associated with an age-dependent increase in bovine fibroblast response to LPS

**DOI:** 10.1186/s12864-017-3796-1

**Published:** 2017-05-25

**Authors:** Filiz T. Korkmaz, David E. Kerr

**Affiliations:** 1 Cellular, Molecular and Biomedical Sciences Program, University of Vermont, 89 Beaumont Avenue, C141C Given, Burlington, VT 05405 USA; 2 Department of Animal and Veterinary Sciences, University of Vermont, 570 Main Street, 213 Terrill Hall, Burlington, VT 05405 USA

**Keywords:** DNA Methylation, Innate Immunity, LPS, Inflammation, RRBS

## Abstract

**Background:**

Differences in DNA methylation are known to contribute to the development of immune-related disorders in humans but relatively little is known about how methylation regulates immune function in cattle. Utilizing whole-transcriptome analyses of bovine dermal fibroblasts, we have previously identified an age and breed-dependent up-regulation of genes within the toll-like receptor 4 (TLR4) pathway that correlates with enhanced fibroblast production of IL-8 in response to lipopolysaccharide (LPS). Age-dependent differences in IL-8 production are abolished by treatment with 5-aza-2-deoxycytidine and Trichostatin A (AZA-TSA), suggesting epigenetic regulation of the innate response to LPS. In the current study, we performed reduced representation bisulfite sequencing (RRBS) on fibroblast cultures isolated from the same animals at 5- and 16-months of age to identify genes that exhibit variable methylation with age. To validate the role of methylation in gene expression, six innate response genes that were hyper-methylated in young animals were assessed by RT-qPCR in fibroblasts from animals at different ages and from different breeds.

**Results:**

We identified 14,094 differentially methylated CpGs (DMCs) that differed between fibroblast cultures at 5- versus 16-months of age. Of the 5065 DMCs that fell within gene regions, 1117 were located within promoters, 1057 were within gene exons and 2891 were within gene introns and 67% were more methylated in young cultures. Transcription factor enrichment of the promoter regions hyper-methylated in young cultures revealed significant regulation by the key pro-inflammatory regulator, NF-κB. Additionally, five out of six chosen genes (*PIK3R1, FES, NFATC1, TNFSF13* and *RORA*) that were more methylated in young cultures showed a significant reduction in expression post-LPS treatment in comparison with older cultures. Two of these genes, *FES* and *NFATC1,* were similarly down-regulated in Angus cultures that also exhibit a low LPS response phenotype.

**Conclusions:**

Our study has identified immune-related loci regulated by DNA methylation in cattle that may contribute to differential cellular response to LPS, two of which exhibit an identical expression profile in both low-responding age and breed phenotypes. Methylation biomarkers of differential immunity may prove useful in developing selection strategies for replacement cows that are less susceptible to severe infections, such as coliform mastitis.

**Electronic supplementary material:**

The online version of this article (doi:10.1186/s12864-017-3796-1) contains supplementary material, which is available to authorized users.

## Background

The innate immune response is an organism’s first line of defense against pathogenic microorganisms and the primary response to tissue damage. Innate immunity is mediated by specialized leukocytes and cells that serve as a barrier to the environment, such as epithelial cells and dermal fibroblasts. Innate immune cells are characterized by their expression of germ-line encoded pattern recognition receptors (PRRs) which recognize conserved molecular patterns (PAMPs), such as lipopolysaccharide found on the outside of Gram-negative bacteria and viral nucleic acids, and elicit the appropriate defense response upon recognition. The most thoroughly characterized of these PRRs are Toll-like receptors (TLRs), a family of type-I transmembrane proteins first discovered in *Drosophila melanogaster* when the *Toll* gene was found to be essential for bacterial and fungal defenses [1, 2]. Upon TLR-ligand binding, an intracellular signaling cascade is activated which allows for the transcription of numerous pro-inflammatory proteins, such as IL-6, TNF-α and IL1-β, through the action of transcription factors NF-κB, AP-1 and Interferon Response Factors (IRFs), among others [3]. While it is clear that the response elicited by TLRs and other PRRs are crucial to the defense against a variety of bacterial, viral and eukaryotic pathogens [4–6], an exacerbated or dysregulated innate response also contributes to infection related inflammatory disorders, such as in antibody dependent enhancement and the subsequent “cytokine storm” characteristic of Dengue hemorrhagic fever [7]. In addition, the type I interferon response, normally beneficial to the host during a viral infection, has been shown to actually increase susceptibility to some bacterial, viral and protozoan infections [8]. The innate response is also a key contributor to a number of sterile pro-inflammatory conditions, such as rheumatoid arthritis and asthma [9, 10].

The severity of bovine mastitis, a primary cause of economic losses to dairy milk production, is similarly more often associated with increased production of pro-inflammatory mediators such as, TNF-α, IL1-ß and IL-6, than it is with number of bacteria present within the mammary gland. Mastitis severity has also been shown to vary greatly, even under controlled experimental settings where pathogen factors are held constant. This suggests host factors play a significant role in mastitis outcome [11]. Several studies have shown a dysregulated population of milk and serum neutrophils, along with greater concentrations of TNF-α and reactive oxygen species, during the peri-partum period when animals are highly susceptible to severe mastitis [12]. Interestingly, priming of the mammary gland with 1 μg of LPS, which causes a substantial but short lived inflammation, reduces severity of experimentally induced *Escherichia coli* (*E. coli)* mastitis 10 days post LPS priming [13]. In an effort to explain the mechanism behind this reduction in mastitis severity, a study by Gunther et al. [14] measured gene expression in response to heat killed *E. coli* in primary mammary epithelial cells following pre-treatment with LPS. Priming of cells was found to increase expression of ß-defensins while decreasing pro-inflammatory and apoptotic gene expression, including IL1-ß, TRAF6, and TNF superfamily proteins (TNFSF13B and TNFSF10), indicating that the previously measured benefit of LPS priming in an intra-mammary infection with *E. coli* was due in part to a reduction in inflammatory response [14]. In a murine mastitis model, administration of LPS into the mammary gland of *TLR4*(−/−) mice to induce mastitis showed that while knockout mice had significantly lower serum cytokines CXCL1, IL1-ß, IL-6, IL-10, TNF-α, CCL2 and IP10, they also experienced a lower degree of mammary gland involution and maintained increased capacity for milk production as compared to wild-type littermates [15]. In agreement with aforementioned studies, our lab has shown that high responding dairy animals, based on dermal fibroblast IL-8 production in response to TLR ligands, have greater tissue damage and neutrophil influx into the mammary gland post-infection and a slower return to pre-infection milk production levels in response to both *Staphylococcus aureus* (*S. aureus*) and *E. coli* mastitis, without any benefit to their ability to clear bacteria [16, 17].

Several factors, including genetic differences, may contribute to variation within an animal’s infection response phenotype. In one example, single nucleotide polymorphisms in the *TLR4* gene have been linked to increased susceptibility to *Mycobacterium avium subspecies tuberculosis*, or Johne’s disease, in Canadian Holsteins [18]. However, a recent study between 3 breeds indicated an estimated heritability of clinical mastitis to be between 2 and 4%, suggesting a very large role for non-genetic factors [19]. Epigenetic modifications, such as DNA methylation, may help to explain discrepancies measured between large phenotypic differences with little genetic basis. In agreement with this, DNA methylation in two breeds of chicken has been shown to contribute to differential susceptibility to infectious diseases such as avian flu and *Salmonella.* Whole genome bisulfite sequencing (WGBS) revealed greater than 5000 differentially methylated regions (DMRs) in lung tissue between the two breeds, with a portion of DMRs falling within gene regions. The methylation data generated by WGBS together with mRNA expression data generated by RNA-seq, also identified genes with both differential methylation and expression, most notably *TLR4* and *PIK3CD* [20].

In support of epigenetic regulation of the bovine innate immune response, data generated in our lab has shown that an age-dependent increase in dermal fibroblast response to LPS is ameliorated following treatment with a DNA methylation and histone deacetylase inhibitor, AZA-TSA [21]. A subsequent study was done to identify methylation and gene expression differences in fibroblast cultures isolated from female calves at 5- and 16-months of age. Transcriptome-wide analysis of these two sets of cultures identified numerous genes within the TLR4 response pathway that were up-regulated in older, more responsive cultures, including TLR4, CD-14, IL-8 and TNF-α [22]. In the same study, we sought to identify changes in DNA methylation between the young and old cultures that contribute to age-related differences in innate immunity, however, the MIRA (methylated CpG island recovery assay)-seq technique that was utilized does not have the resolution required to determine methylation differences at the base pair level. As such, very little difference in methylation was found between the two sets of cultures.

Another phenotypic difference we have identified is the differential response between Holstein and Angus breeds, where Holstein cattle exhibit a much higher fibroblast response to LPS than Angus cattle. The few reports of mastitis in beef cows have also suggested beef breeds are less susceptible to coliform mastitis, with no cases of *E. coli* mastitis cases detected in several studies [23, 24]. In this case, genetic differences between breeds are likely to have an influence, however, calves from these two breeds experience highly disparate upbringing. In contrast with Angus and other beef breeds, Holstein calves are immediately separated from their dam allowing for little to no maternal care which has been shown to contribute to differential methylation of hormone receptors in rats [25]. Again, transcriptome analysis of age-matched fibroblasts isolated from the two breeds revealed a number of pro-inflammatory genes with differential expression, with Holstein cultures exhibiting significant up-regulation as compared to Angus cultures. However, little to no difference in DNA methylation was found using the MIRA-seq technique [26].

Knowledge of epigenetic regulation of the immune response in dairy cattle is currently lacking and will be important to understanding differences in mastitis susceptibility. From the previous studies, it is clear that methylation differences at a base pair resolution are required. As such, the aim of the current study was to identify base-pair specific DNA methylation differences in fibroblasts taken from Holstein heifers at 5- and 16-months of age using reduced representation bisulfite sequencing (RRBS). Once candidate differentially methylated genes were identified, we determined whether methylation effects gene expression by measuring fibroblast gene expression at various time points post-LPS treatment. Furthermore, we determined whether genes regulated by methylation in young and old cultures also differed in gene expression between Angus and Holstein cultures. Overall the current study contributes to our knowledge of candidate immune response genes that are subject to regulation by DNA methylation within the genome of dairy cattle and may lead to a better understanding of the mechanisms that regulate inter-animal variation in susceptibility to severe mastitis.

## Methods

### Experimental animals

Six pairs of dermal fibroblast samples, collected from Holstein heifers at 5 and 16 months of age, were used from a previously characterized cohort of 15 heifer fibroblast samples [27]. Primary dermal fibroblast collection and ranking based on IL-8 production following in vitro LPS stimulation was previously described [27]. The six pairs of fibroblast samples chosen for DNA methylation analysis were all selected from mid-responding animals to reduce inter-animal variation within the two groups that could potentially interfere with any true age effects.

A second set of dermal fibroblasts was collected for the breed difference experiments. Fibroblasts were isolated from four 19-month Holstein and four 19-month Black Angus cows. Fibroblast collection, isolation and LPS response are described elsewhere [26].

In both the age and breed experiments, fibroblasts were stimulated with 100 ng/ml of LPS and RNA isolation was performed side-by-side in neighboring wells. From the same vial of cryopreserved cells used in the LPS challenge, some cells were cultured in a T-75 cm^2^ culture flask for DNA isolation. Cells were again cultured side-by-side and untreated cultures were used for DNA extraction.

All experiments were approved by the Institutional Animal Care and Use Committee at the University of Vermont.

### Fibroblast culturing and LPS challenge

Growth media for fibroblast cultures was Dulbecco’s Modified Eagle Media (DMEM; Hyclone Laboratories, Logan, UT) supplemented with 5% fetal bovine serum (FBS; Hyclone Laboratories), 1% penicillin-streptomycin (Hyclone Laboratories) and 1% insulin-transferrin-selenium (ITS; Mediatech Inc., Herndon, VA). Fibroblasts were revived from cryopreservation and expanded in a T-75 cm^2^ culture flask at 37 °C 5% CO_2_ in a humidified incubator. Confluent flasks were then treated with 0.25% trypsin (MP Biomedical, Santa Ana, CA) and cells were transferred to a 6-well plate at 1.25 × 10^5^ cells/ml in a total volume of 2 ml, or 2.5 × 10^5^ total cells and cultured for 24 h for the LPS challenge experiments. Remaining cells were transferred to a T-75 cm^2^ culture flask and allowed to grow to confluency for subsequent DNA isolation.

After 24 h, cells in the 6-well plates were treated with 100 ng/ml of ultra-pure LPS isolated from *Escherichia coli* O111.B4 (Sigma-Aldrich, St. Louis, MO) for either 0, 2, 8 or 36 h. At 0 and 36 h, media was collected for protein production analysis. After removal from the wells, media was centrifuged at 10,000 × g for 1 min to remove cellular debris and stored at −20 °C until further analysis. At every time point, cells were rinsed gently with Dulbecco’s Phosphate Buffered Saline (DPBS; Hyclone Laboratories) and cell lysate was collected by adding 500 μl cell lysis buffer (5 Prime, Hamburg, Germany) to the well. Cell lysate was stored at −20 °C until RNA isolation.

Cells that were grown in a T-75 cm^2^ culture flask for DNA isolation were treated with trypsin at confluency and centrifuged at 400 × g. The cell pellet was then lysed with 300 μl cell lysis buffer (5 Prime) by adding the lysis buffer and vortexing for 15 s. The cell lysate was stored at −20 °C until DNA isolation.

### Quantification of IL-8 and IL-6 protein

Interleukin-8 production in media from LPS stimulated dermal fibroblasts was determined with a sandwich ELISA (Mabtech Inc., Cincinnati, OH) per manufacturer’s protocol with slight modifications. Capture antibody was diluted 1:500 to 1 μg/ml in 0.05 *M* bi-carbonate buffer. Recombinant bovine IL-8 (Thermo Scientific, Rockford, IL) was used as the assay standard with a detection limit of 156.25 pg/ml. Capture antibody was diluted 1:20,000 in PBS-0.05% Tween-20 to a concentration of 0.025 μg/ml. Streptavidin-horseradish peroxidase was diluted 1:15,000 in PBS-0.05% Tween-20 to a concentration of 0.07 μg/ml.

Interleukin-6 production was also determined by a sandwich ELISA (Thermo Scientific) per manufacturer’s instructions. Briefly, capture antibody was diluted 1:100 in 0.05 *M* bi-carbonate buffer. Recombinant bovine IL-6 (Thermo Scientific) was used as the assay standard with a detection limit of 156.25 pg/ml. Detection antibody was diluted 1:100 in PBS-0.1% BSA. Streptavidin-horseradish peroxidase (Sigma-Aldrich) was diluted 1:2000 in PBS-0.1% BSA to a concentration of 0.5 μg/ml. Development of IL-8 and IL-6 ELISA plates was done by adding 3,3’,5,5’-tetramethylbenzidine substrate (TMB; Thermo Scientific) to the wells and the reaction stopped with 1 *M* H_2_SO_4_.

### DNA extraction and preparation for RRBS

DNA was extracted from 5- and 16- month dermal fibroblasts using a 5-Prime PerfectPure^TM^ Archive DNA Extraction Kit (5 Prime) per manufacturer’s protocol. DNA concentrations were then determined using a Qubit^TM^ 2.0 Spectrofluorometer (Life Technologies, Carlsbad, CA). DNA was diluted to 40 ng/μl and a total of 2 μg was sent to Zymo Research (Irvine, CA) for Methyl-MiniSeq^TM^ RRBS library preparation. Libraries were generated with 200–500 ng of DNA as previously described [28]. Briefly, DNA was sequentially digested with 60 units of TaqαI and 30 units of *MSP*I (NEB, Ipswich, MA) which recognizes CCGG as a cut site and cleaves after the first cytosine, creating DNA products with CG dinucleotides on both ends of the DNA and enriching for CG rich regions. Following enzymatic digestion, DNA products were end-repaired, A-tailed and extracted with DNA Clean and Concentrator Kit^TM^ (Zymo Research). The extracted DNA was then ligated to methylated Illumina primers using the Illumina DNA preparation kit and protocol (Illumina, San Diego, CA). Adaptor ligated DNA was then size selected for desired input fragments (150–250 bp and 250–350 bp) with a 2.5% NuSieve 3:1 agarose gel and extracted using the Zymoclean^TM^ Gel DNA Recovery Kit (Zymo Research). Successfully ligated and purified DNA was then bisulfite converted using the EZ DNA Methylation-Lightning^TM^ Kit (Zymo Research). Control DNA was similarly bisulfite converted to assess conversion rates, which were 99% for all samples. Following conversion, preparative-scale PCR was performed with a total of 16 cycles and PCR products were purified with the DNA Clean and Concentrator Kit^TM^ (Zymo Research). DNA libraries were then sequenced on the Illumina HiSeq2000 (Illumina), generating 50 bp paired-end reads and base-calling was performed using standard Illumina base-calling software.

### DNA sequence processing and alignment

Following sequencing, bioinformatics analysis was performed at Zymo Research using a proprietary analysis pipeline written in Python. Prior to alignment, reads were assessed for quality using FastQC (v0.11.1, Babraham Bioinformatics, UK) and Trim Galore (v0.3.7, Babraham Bioinformatics, UK). Bases with a Phred score greater than 20 were kept for downstream analysis (−−*paired --phred33 -q 20)*. Illumina adaptor trimming was done using Trim Galore with the default settings which are automatically set to standard Illumina adaptors unless otherwise specified. Trim Galore also contains a setting specifically for trimming RRBS data (−−*rrbs*) which was used to further modify our reads. RRBS introduces artificial CpG sites which require trimming in order to avoid them being used in methylation calling. To do so, Trim Galore trims the first 2 bases from the 3′ end of the sequence so the C closest to the second enzyme cut site is not included in methylation calling. Reads were then mapped to an *in-silico* bisulfite converted reference genome (BosTau8/BTau_4.6.1) with the Babraham Bismark software (v0.13.1, Babraham Bioinformatics, UK) using the *bismark_genome_preparation* command, the entire reference genome and with the --*non_directional* parameter applied. Babraham Bismark is designed for aligning bisulfite sequencing data while simultaneously making methylation calls. In the process of bisulfite sequencing, un-methylated cytosines are converted to uracil, while methylated cytosines are unaffected. The result of this is four sets of potential sequences at any given locus. To determine which of these four potential sequences is correct, Bismark creates two bisulfite converted reference genomes, one that is a C ➔ T conversion and one that is a G ➔ A conversion to account for conversion on the reverse strand. Each sequence read is also bisulfite converted *in silico* and is aligned to the pre-converted version of the reference genome. The best alignment is then identified and used to make a methylation call. Alignments that are uniquely mapped were kept for analysis. Alignments that mapped to multiple regions were discarded. Due to enrichment-constraints of RRBS libraries, duplicates measured as a result of shorter, overlapping paired end inserts were not removed in the sequence data and were counted as two reads.

On average, mapping efficiencies were 25% and ranged between 23 and 27% and the average number of unique CpGs identified were 6.0 million and ranged between 4.8 and 6.6 million. Differentially methylated CpGs falling within −2.5 kb, +1.0 kb of an annotated transcription start site (TSS) were defined as being within the promoter region of a gene. Methylation ratios were used as the comparison parameter to test statistical differences in DNA methylation and these were defined as the number of reads overlapping a particular CpG site which contained either a cytosine or thymine nucleotide. Ratios were then calculated as Methylation Ratio (Mr) = (C)/(C + T).

### Functional analysis

KEGG (Kyoto Encyclopedia for Genes and Genomes) pathway, GO (Gene Ontology) and UCSC (University of California Santa Cruz) Transcription Factor Binding Site (TFBS) analyses were performed using the DAVID (the Database for Annotation, Visualization, and Integrated Discovery) 6.8 platform [29, 30]. Four lists of official gene IDs (all IDs or promoter only with ≥5× or ≥10× coverage) were generated for sites located within annotated genes that were hyper-methylated in either young or old cultures (Additional files 1 and 2). A suggestive analysis was performed on all differentially methylated genes with ≥5× coverage and a conserved analysis was performed with only genes that had ≥10× coverage. Gene IDs from all three gene regions (exon, intron and promoter) were inputted into DAVID, converted to Entrez gene IDs and default parameters were used for KEGG and GO analyses. Only those genes that were covered by RRBS, either at the 5× or 10× level, were set as the background. Promoter gene IDs were used in transcription factor enrichment analysis and TFBS analysis was similarly run with default parameters and *Homo sapiens* as the background due to a lack of support for *Bos taurus* within the UCSC_TFBS tool.

### Gene expression of selected genes

Selected differentially methylated genes and LPS response genes were measured in all fibroblast cultures exposed to LPS for 0, 2 and 8 h. RNA was isolated using the 5 Prime PerfectPure^TM^ RNA Cultured Cell Kit (5 Prime) per manufacturer’s instructions and quantified using a Qubit^TM^ 2.0 Fluorometer (Life Technologies, Carlsbad, CA). First strand cDNA synthesis was done using the ImpromII^TM^ Reverse Transcriptase Kit (Promega, Madison, WI). To determine gene expression, quantitative reverse transcriptase PCR (RT-qPCR) was performed using Thermo Scientific™ Maxima™ SYBR™ Green/Fluorescein 2× qPCR Master Mix (Thermo Fisher) on a CFX96 Touch™ Real-Time PCR machine (Biorad, Hercules, CA) on the selected genes. The gene β-Actin was used as a housekeeping gene control. Cycling conditions were as follows: 95 °C for 2 min followed by 40 cycles of 95 °C for 15 s, 60 °C for 1 min and 72 °C for 1 min after which a melt curve was inserted. All oligonucleotide primer sequences are shown in Table 1. Unless already published primers were available, all primers were designed using Primer 3 on NCBI. An amplicon of 200 – 300 base pairs was designed and the primer binding site could not contain any known SNP. Each primer sequence was then analyzed with an NCBI nucleotide BLAST search to ensure only the intended *Bos taurus* gene was detected. Melt curve analysis was performed on each primer pair and included a negative control reaction (no cDNA). Melt curve analysis revealed only one peak indicative of a single product with no peaks associated with the negative control.

**Table 1:** Real time PCR primers

Gene name	Sequence 5′ to 3′	Reference
β-Actin	F: GCAAATGCTTCTAGGCGGACT R: CAATCTCATCTCGTTTTCTGCG	[84]
Feline Sarcoma Oncogene (FES)	F: GTCTCAGACAAGTCCCCGTG R: AGTCTGAACACAGCGTCAGG	Designed in house
Interleukin 6 (IL6)	F: TGAGGGAAATCAGGAAAATGT R: CAGTGTTTGTGGCTGGAGTG	[84]
Interleukin 8 (IL8)	F: GCTGGCTGTTGCTCTCTTG R: AGGTGTGGAATGTGTTTTTATGC	[84]
Nuclear factor of activated T-cells calcineurin dependent 1 (NFATc1)	F: GTCCGACGTCAAGCGGTAG R: TTGACCGTTACGGGAATGGG	Designed in house
Phosphatidylinositol 3-kinase regulatory subunit alpha (PIK3R1)	F: TCATTCCGGTAGCCGTTTCC R: CTCAGAACTTGCTGCTGGGA	Designed in house
RAR-Related Orphan Receptor A (RORA)	F: ATAACATCTCGGCCAACGGG R: GGAAGAAGCCTGATGCTGGT	Designed in house
Transcription factor 7 (T-cell specific, HMG-box) (TCF7)	F: GAGCCAAAGTCATTGCGGAG R: TCTTTTTCCTCCTGAGTTGGATTC	Designed in house
Tumor Necrosis Factor Super Family 13 (TNFSF13)	F: AGAAGCGCTCAGTTCTGCAT R: CTGTTGTAGGCCCAGTCAGG	Designed in house

### Statistical analysis

To assess differences in cytokine protein production a paired Student’s *t* test was performed using GraphPad Prism Version 6.0 for Windows (GraphPad Software, La Jolla, CA). Differences in DNA methylation at adequately covered (≥5× coverage based on similar RRBS studies [31, 32]) CpG sites was performed at Zymo Research and determined using a paired Student’s *t* test comparing methylation ratios at each site. A clustering dendogram was made according to methylation ratio on the top 100 differentially methylated sites, based on *p*-value. The clustering analysis was performed at Zymo Research using a standard data analysis algorithm with a “complete” linkage method and a “Euclidean Distance” metric. Significance for GO, KEGG and TFBS were determined by a modified Fisher’s Exact test in DAVID as previously described [29] and multiple comparisons correction were done using the Benjamini-Hochberg method. Gene expression differences were determined using either a paired (young vs. old) or unpaired (Angus vs. Holstein) Student’s *t* test at each time point in GraphPad. Protein concentrations and delta Ct values are expressed as the mean +/− standard error (SEM).

## Results

### Fibroblast cytokine response to LPS

**Fig. 1:**
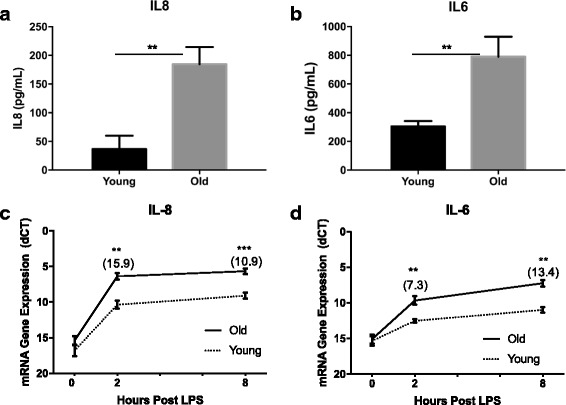
Fibroblast Response to LPS in Young versus Old Cultures. Interleukin-8 (**a & c**) and Interleukin-6 (**b & d**) protein production and gene expression were measured in young and old dermal fibroblasts (n = 6 per group) at various time points post LPS. Protein production is presented in pg/ml following 36 h of LPS stimulation. Gene expression was measured by RT-qPCR and is presented as the change in cycles to threshold (dCT) of gene expression at 0, 2 & 8 h post LPS in comparison to β-actin. Fold change gene expression (Old > Young) is presented in parentheses above each significant time point. All values are displayed as mean (+/− SEM). Significance was measured using a paired Student’s t test at each time point and ** = *P* < 0.01 *** = *P* < 0.001

Dermal fibroblasts from the same six animals taken at 5 and 16 months of age were revived from cryopreservation for a total of 12 cultures (6 young vs. 6 old). Cultures were subsequently stimulated with LPS (100 ng/ml) for 36 h to determine cytokine protein concentration in the media. Media from unstimulated cultures was used as a negative control. Protein concentrations of interleukin-8 (IL-8) are shown in Fig. 1, panel a. As expected, the 16-month cultures produced significantly more IL-8 (*P* < 0.01) than the 5-month cultures. At 36 h, older cultures had an approximately 5-fold higher concentration of IL-8 than young cultures. No detectable IL-8 was produced in media only controls. Interleukin-6 (IL-6) was also measured in media post 36-h LPS stimulation. In line with IL-8 protein, 16-month cultures produced significantly more IL-6 (*P* < 0.01) than 5-month cultures (Fig. 1, panel b). This difference in older cultures was about 2.5-fold more IL-6 than young cultures. Again, control media IL-6 levels fell below detection limits of this assay.

Next, to determine if cytokine gene expression was also different between the young and old cultures, RT-qPCR was performed on fibroblasts at 0, 2 and 8 h post LPS stimulation. In line with protein production, IL8 and IL6 gene expression levels were upregulated (*P* < 0.01) in old versus young cultures (Fig. 1, panels c and d). Older fibroblast cultures had a 15.9 and 10.9-fold increase in IL8 gene expression and a 7.3 and 13.4-fold increase in IL6 gene expression over younger cultures at 2 and 8 h post LPS stimulation, respectively.

Cytokine protein and expression analyses confirmed that the older dermal fibroblast cultures were much more responsive to LPS than younger cultures. Genomic DNA was then isolated from unstimulated cultures, grown from the same cryopreserved stock used to assess LPS response, and analyzed by RRBS to determine whether methylation differences may contribute to differences in the LPS-induced response.

### Reduced representation bisulfite sequencing in 5-month and 16-month dermal fibroblasts

**Fig. 2:**
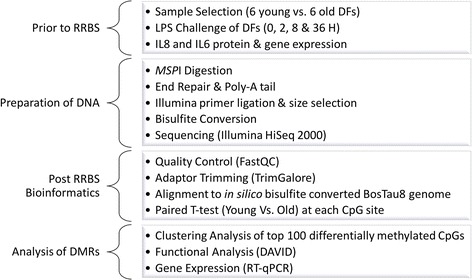
RRBS Project Workflow. Prior to methylation analysis, 6 pairs of fibroblasts isolated from the same animals at 5- and 16-months of age were selected and challenged with LPS for 0, 2, 8 and 36 h to measure cytokine protein and gene expression. In preparation for RRBS, fibroblast DNA was digested with *MSP*I to enrich for CpG rich regions of the genome, followed by end-repair and Poly-A tail addition. The digested DNA was then ligated to Illumina primers, size selected and bisulfite converted prior to sequencing. The subsequent reads were quality controlled by FastQC and Illumina adaptors were trimmed with TrimGalore. Those that passed quality control were then aligned to the BosTau8 genome and uniquely mapped reads were kept for analysis with a paired T test at each CpG site with greater than or equal to 5× coverage. Once differentially methylated sites were identified, clustering analysis was performed on the top 100 sites based on *p*-value. Functional analysis to identify enriched KEGG pathways, GO terms and transcription factors was performed by DAVID and gene expression analysis by RT-qPCR

As outlined in the RRBS project workflow in Fig. 2, genomic DNA from all the cultures were treated with a methylation insensitive restriction enzyme *MSP*I. The restriction enzyme cuts at CCGG sites, following the first cytosine, yielding CG sites on both ends of the resulting DNA fragments. This results in enrichment of fragments from regions in the genomic DNA with many CpG sites, such as CpG islands, that are typically associated with promoter regions of DNA [33]. In our analysis, 65% of annotated promoter regions, defined as −2500 to +1000 bp from the transcription start site (TSS) had greater than 50× coverage, 8% had less than 50× but greater than 10× coverage, 4% had less than 10× coverage and 23% were not represented.

Following bisulfite conversion, sequencing and post-sequencing bioinformatics, a paired T-test was performed on the methylation ratios between young and old cultures at each sequenced CpG site that met coverage requirements. Methylation analysis revealed 14,094 differentially methylated CpG sites with *P* < 0.05 and at least 5× coverage. Of these, 9351 (67%) were more methylated in young cultures and 4743 (33%) were more methylated in old cultures. Clustering analysis was performed on the top 100 differentially methylated sites (i.e. sites with the greatest significant difference in methylation ratios). The old cultures and young cultures clustered separately, indicating that two distinct methylation profiles exist, where young cultures form one profile and old cultures form a second (Additional file 3).

### Suggestive analysis of differentially methylated genes

Next, we performed a comprehensive, but suggestive functional analysis of all the sites that fell within gene regions and had ≥5× coverage. Of the 9351 sites more methylated in young cultures, 752 regions were located in annotated promoters, 720 in gene exons and 1926 in gene introns. Of the 4743 sites more methylated in old cultures, 365 were located in annotated promoters, 337 in gene exons and 965 in gene introns. Sites that fell within annotated genes were used to generate lists of gene IDs with greater methylation in either young or old cultures and functional analysis was performed using DAVID. KEGG pathway analysis on genes more methylated in young cultures identified 5 significantly enriched pathways. These included the “Adherens Junction”, “Proteoglycans in Cancer”, “Rap1 Signaling Pathway”, “Pathways in cancer” and “Melanogenesis”. It is worth noting that the “PI3K-Akt Signaling Pathway” was also enriched with *P* = 0.06 after multiple comparisons correction. In older cultures, 16 pathways were significantly enriched, with the “Cholinergic Synapse” pathway as the top result. A number of secretory pathways and hormone synthesis pathways were also significantly enriched. Among the 16 pathways, those that were of most interest to the current study were the “Calcium Signaling Pathway”, “Inflammatory mediator regulation of TRP channels” and “cAMP signaling pathway”. Among the top significantly enriched GO terms in genes with greater methylation in young cultures were “Transcription Repressor Activity”, “Sequence Specific DNA Binding”, and “RNA Polymerase II regulatory region sequence-specific DNA binding”. Interestingly, in older cultures, only one GO term, “Plasma Membrane” was significant and, in contrast to young cultures, lacked terms related to regulation of gene transcription.

Using the annotated genes from only the promoter sites with greater methylation in either young or old cultures, a transcription factor enrichment analysis was performed using DAVID and *Homo sapiens* as the background species, because *Bos taurus* is not yet supported in the UCSC_TFBS function. Table 2 shows the top 15 most significantly enriched transcription factors that regulate the inputted gene promoters more methylated in young cultures and Table 3 shows transcription factors enriched in gene promoters more methylated in old cultures. A number of transcription factors associated with immune system regulation and the inflammatory response were associated with promoters more methylated in both young and old cultures. Most noteworthy in young cultures was greater methylation in NF-κB associated genes. Also, significantly more methylated in young cultures were genes regulated by PAX5 (also known as B-cell activating protein), and CREB (cAMP-regulated binding protein), a phosphorylation responsive transcription factor that binds to cAMP responsive elements.

**Table 2:** Suggestive transcription factor enrichment of gene promoters more methylated in young cultures

Category	Term	Count^a^	Fold enrichment^b^	FDR^c^
UCSC_TFBS	PAX5	288	1.32	4.35E-09
UCSC_TFBS	LMO2COM	297	1.31	6.04E-09
UCSC_TFBS	P53	321	1.25	2.23E-08
UCSC_TFBS	EGR3	111	1.77	5.34E-08
UCSC_TFBS	AP2	148	1.57	1.04E-07
UCSC_TFBS	NMYC	174	1.48	1.57E-07
UCSC_TFBS	ARNT	243	1.33	2.26E-07
UCSC_TFBS	NRSF	265	1.29	3.42E-07
UCSC_TFBS	MYCMAX	302	1.23	8.19E-07
UCSC_TFBS	NFKB	246	1.30	1.43E-06
UCSC_TFBS	AHRARNT	259	1.28	1.49E-06
UCSC_TFBS	CREB	218	1.33	1.79E-06
UCSC_TFBS	E47	290	1.23	2.99E-06
UCSC_TFBS	COUP	209	1.33	5.73E-06
UCSC_TFBS	TAXCREB	262	1.25	6.31E-06

^a^Number of inputted genes regulated by transcription factor listed

^b^Fold enrichment as measured by Fisher’s Exact Test

^c^Benjamini-Hochberg False Discovery Rate

**Table 3:** Suggestive transcription factor enrichment of gene promoters more methylated in old cultures

Category	Term	Count^a^	Fold enrichment^b^	FDR^c^
UCSC_TFBS	PAX5	187	1.43	8.40E-10
UCSC_TFBS	SP1	95	1.73	1.66E-06
UCSC_TFBS	ATF	113	1.58	3.02E-06
UCSC_TFBS	NRSF	165	1.35	9.59E-06
UCSC_TFBS	MIF1	136	1.43	1.06E-05
UCSC_TFBS	P300	124	1.47	1.09E-05
UCSC_TFBS	ZID	150	1.38	1.14E-05
UCSC_TFBS	SREBP1	178	1.28	4.10E-05
UCSC_TFBS	MYOGNF1	145	1.35	6.27E-05
UCSC_TFBS	TAXCREB	162	1.30	8.37E-05
UCSC_TFBS	ZIC3	123	1.41	8.80E-05
UCSC_TFBS	MZF1	147	1.33	9.46E-05
UCSC_TFBS	AHRARNT	158	1.31	9.57E-05
UCSC_TFBS	HMX1	139	1.34	1.39E-04
UCSC_TFBS	TAL1BETAITF2	158	1.29	1.50E-04

^a^Number of inputted genes regulated by transcription factor listed

^b^Fold enrichment as measured by Fisher’s Exact Test

^c^Benjamini-Hochberg False Discovery Rate

In old cultures, PAX5 was similarly enriched along with SP.1, p300 (a CREB co-activator), and ATF or activating transcription factors, which are diverse members of the ATF/CREB family of transcription factors with a range of physiological functions.

### Conserved analysis of differentially methylated genes

In a second, conserved analysis we focused solely on sites with ≥10× coverage, which is the minimum coverage that is necessary to accurately determine differentially methylated sites in an RRBS analysis. The additional filtering left 2865 differentially methylated sites. Of these, 1922 were more methylated in young cultures and 943 were more methylated in old cultures. In young cultures, 134 gene promoters, 98 exons and 354 introns were hyper-methylated and in old cultures only 42 gene promoters, 45 exons and 150 introns were hyper-methylated in comparison to young cultures. The genes associated with these regions were used to conduct a conserved GO, KEGG and TFBS analysis. Interestingly, with the additional filtering we found no significant GO terms associated with either young or old cultures, although “Semaphorin Receptor Activity” trended (*P* < 0.10) towards significance in old cultures. Two KEGG pathways, “Adherens Junction” and “Proteoglycans in Cancer” remained significantly associated with young cultures and no KEGG pathways were found to be associated with old cultures. Finally, 21 transcription factors were identified as significantly enriched in hyper-methylated promoter regions in young cultures, the top 10 of which are listed in Table 4. We did not identify any transcription factors enriched in our assessment of hyper-methylated promoters in old cultures. Many of the transcription factors that were identified in the suggestive analysis in young cultures remained significant, including PAX5 and most notably, NF-κB that was found to be associated with 56 out of 134 gene promoters more methylated in young cultures.

**Table 4:** Conserved transcription factor enrichment of gene promoters more methylated in young cultures

Category	Term	Count^a^	Fold enrichment^b^	FDR^c^
UCSC_TFBS	P300	49	1.67	0.007
UCSC_TFBS	TAL1BETAITF2	60	1.40	0.024
UCSC_TFBS	CDC5	52	1.46	0.027
UCSC_TFBS	ROAZ	52	1.44	0.027
UCSC_TFBS	P53	69	1.29	0.028
UCSC_TFBS	COUP	48	1.46	0.031
UCSC_TFBS	NFKB	56	1.42	0.032
UCSC_TFBS	EGR3	25	1.92	0.032
UCSC_TFBS	AP2	35	1.78	0.032
UCSC_TFBS	AP2GAMMA	18	2.19	0.040

^a^Number of inputted genes regulated by transcription factor listed

^b^Fold enrichment as measured by Fisher’s Exact Test

^c^Benjamini-Hochberg False Discovery Rate

### Fibroblast gene expression of differentially methylated genes

Selected differentially methylated genes were analyzed by RT-qPCR to determine whether changes in methylation resulted in differences in gene expression. The rationale for choosing the analyzed genes was that there was a greater than 25% methylation difference in at least one region in young versus old cultures, the sites had on average greater than 10× coverage across the 12 animals, methylation was greater in young versus old cultures and that there was some evidence in the literature that the genes are involved in immune response regulation. The six selected genes, and number and location of differentially methylated regions are listed in Table 5, along with a summary of expression differences.

**Table 5:** Summary of selected innate response genes hyper-methylated in young cultures

Gene name	Number of differentially methylated sites^a^	Location of differentially methylated sites	Expression (Old > Young) at 0, 2 & 8 H post LPS^b^	Expression (Holstein > Angus) at 0, 2, & 8 H post LPS^b^
FES	2	Promoter, Exon	5.7, 4.3, 6.3	3.7, 5.4, 4.8
NFATc1	3	Promoter, Intron	1.8, 1.8, ---	2.2, 6.5, 1.7
PIK3R1	2	Promoter, Intron	3.0, 2.6, 2.5	---, 2.4, (−1.9)
RORA	5	Intron	---, ---, 2.1	2.0, 2.2, ---
TNFSF13	1	Promoter	2.0, 1.6, 2.8	---, ---, ---
TCF7	2	Promoter	---, ---, ---	4.5, ---, 4.3

^a^Number of differentially methylated CpGs (*P* < 0.05, >25% meth diff)

^b^Displayed as fold change at 0, 2 or 8 H post LPS. Positive values indicate (Old > Young) or (Holstein > Angus), negative values indicate (Young > Old) or (Angus > Holstein) and --- signifies *P* > 0.10

**Fig. 3:**
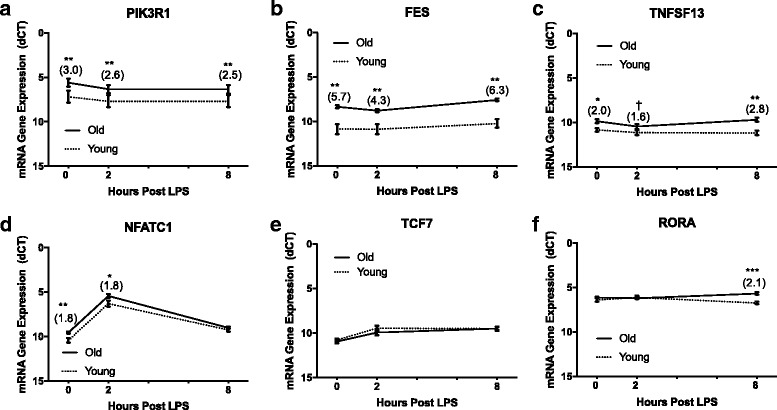
Gene Expression post LPS treatment of Selected Differentially Methylated Genes in Young and Old Fibroblast Cultures. Expression of six genes more methylated in young cultures (**a**) PIK3R1, (**b**) FES, (**c**) TNFSF13, (**d**) NFATC1, (**e**) TCF7 and (**f**) RORA were measured in young and old fibroblasts (*n* = 6 per group) at 0, 2 and 8 h post LPS. Expression was measured by RT-qPCR and values are presented as dCT in comparison to β-actin. Fold change gene expression (Old > Young) is displayed in parentheses above each significantly different time points. All values are displayed as mean (+/− SEM). Significance was determined using a paired Student’s t Test at each time point post LPS treatment and *** = *P* < 0.001, ** = *P* < 0.01, * = *P* < 0.05, † = *P* < 0.10

Gene expression analysis of the LPS responsiveness of the six selected genes hyper-methylated in young versus old cultures is shown in Fig. 3. Overall, 5 of the 6 selected genes showed a reduced expression in cultures established from the animals at 5 vs. 16 months of age. Two of the 6 genes, *FES* and *PIK3R1*, had reduced expression in the younger cultures at all 3 time points, with a 5.7, 4.3 and 6.3-fold (*P* < 0.01) lower expression in FES and 3.0, 2.6, and 2.5-fold (*P* < 0.01) lower expression in PIK3R1 at 0, 2 and 8 h post LPS stimulation, respectively. Both genes had differentially methylated sites located in the promoter region, a single site located 23 base pairs upstream from the transcription start site (TSS) of *PIK3R1* and two sites, 4 and 6 base pairs, downstream of the TSS in *FES*. Additionally, *FES* had two differentially methylated sites located within exon regions and *PIK3R1* had one site located within the first intron of the gene. Expression of these genes was not affected by LPS stimulation.

A gene that encodes a TNF superfamily member ligand, TNFSF13 also known as APRIL, also showed significantly lower expression in young cultures at 0 and 8 h post LPS stimulation (2.0 and 2.8-fold, *P* < 0.05), with a trend towards significantly lower expression at 2 h (1.6-fold, *P* < 0.10), but was not affected by LPS stimulation. *TNFSF13* had one differentially methylated site in the gene promoter region, 145 bases upstream from the TSS in the anti-sense position and directly downstream from a site on the positive strand with 93% sequence similarity to the consensus NF-kB binding site. The gene, *NFATC1*, that encodes an LPS responsive transcription factor, had significantly lower expression in young versus old cultures at 0 and 2 h post LPS stimulation, with both time points showing a 1.8-fold (*P* < 0.05) lower expression in young cultures. Expression of NFATc1 also increased 17.5 +/− 5.6-fold 2 h post LPS stimulation, with no difference in fold change between young and old cultures. By 8 h post-challenge, expression returned to near basal levels. The *NFATC1* gene had one differentially methylated site located within a CpG island in the promoter region, approximately 1000 bp upstream from the TSS and downstream of a potential Sp1 binding site. Two additional differentially methylated sites were located within the last intron of the *NFATC1* gene.

The only selected gene that did not have differences in promoter methylation, *RORA*, had 6 differentially methylated sites located in the first intron of the gene. Gene expression analysis showed significantly lower levels of expression in young versus old cultures at 8 h post LPS treatment (2.1-fold, *P* < 0.001) with no change in response to LPS. Finally, we measured *TCF7*, the gene that transcribes the transcription factor TCF7, that had 2 differentially methylated sites located within a CpG island in the promoter region. The first CpG was located approximately 1200 bp upstream from the TSS and near to a potential NFAT5 binding site and the second CpG was approximately 979 bp upstream from the TSS on the anti-sense strand. Interestingly, no significant expression differences were detected in young versus old cultures. However, there was a small increase in gene expression of TCF7 in response to LPS. Expression was increased 2.3 +/− 0.7-fold and 2.7 +/− 0.9-fold at 2 and 8 h, respectively, with no difference between young and old cultures.

### Gene expression of differentially methylated genes in Angus vs. Holstein fibroblast cultures

Finally, we wanted to determine whether differentially methylated genes identified in young versus old cultures would differ in expression in a second high vs. low LPS responsive cattle phenotype recently discovered in our laboratory. Just as with the age difference, we have shown that dermal fibroblasts collected from the Holstein breed are much more responsive to LPS than fibroblasts collected from the Angus breed [26]. To determine whether similar gene expression differences could be detected between breeds, RT-qPCR was performed on age-matched dermal fibroblast cultures at 0, 2 and 8 h post-LPS treatment. Greater LPS-induced IL-8 and IL-6 protein production and gene expression in Holstein vs. Angus cultures have been described elsewhere [26].

**Fig. 4:**
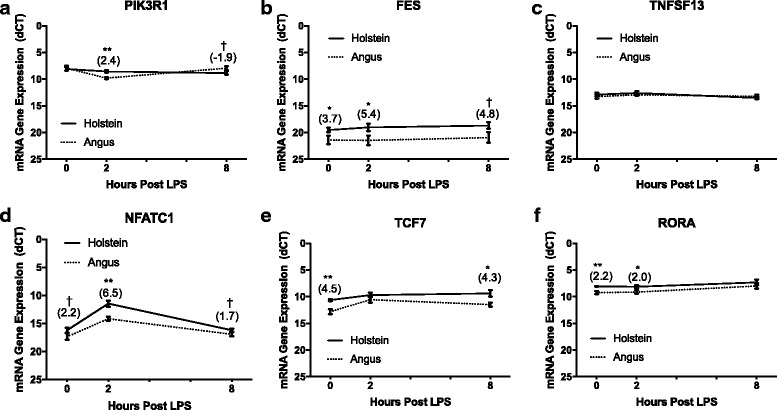
Gene Expression post LPS Treatment of Selected Genes from RRBS Analysis in Angus and Holstein Fibroblast Cultures. Expression of six genes more methylated in young cultures, (**a**) PIK3R1, (**b**) FES, (**c**) TNFSF13, (**d**) NFATC1, (**e**) TCF7 and (**f**) RORA were measured in fibroblasts isolated from 19-month Angus and Holstein cows (n = 4 per group) at 0, 2 and 8 h post LPS. Expression was measured by RT-qPCR and values are presented as dCT in comparison to β-actin. Fold change gene expression is displayed in parentheses above each significantly different time point, whereby positive values indicate Holstein > Angus and negative values indicate Angus > Holstein. All values are displayed as mean (+/− SEM). Significance was determined using an unpaired Student’s t Test at each time point post LPS treatment and ** = *P* < 0.01, * = *P* < 0.05, † = *P* < 0.10

Gene expression analysis was performed on the same 6 genes that were differentially methylated in the young versus old fibroblast cultures, where young cultures had greater methylation and, with the exception of TCF7, lower expression. As shown in Fig. 4 and summarized in Table 5, the majority of the genes were similarly upregulated in Holstein cultures as they were in older cultures. Two notable exceptions to the pattern seen in our young versus old analysis were in TNFSF13, where no difference was measured in Angus versus Holstein cultures, and in TCF7 which was upregulated in Holstein cultures at 0 and 8 h post LPS treatment. Under basal conditions, Holstein cultures expressed TCF7 4.5-fold (*P* < 0.01) higher than Angus cultures and at 8 h post LPS TCF7 expression was 4.3-fold (*P* < 0.05) higher in Holstein cultures. Interestingly, at 2 h post LPS treatment, Angus cultures increased TCF7 gene expression 5-fold, while Holstein cultures only increased expression 2-fold. This fold increase at 2 h was significantly higher in Angus cultures (*P* < 0.05) as compared to Holstein cultures, although Holstein cultures did have a significantly (*P* < 0.05) higher level of expression at 2 versus 0 h. While Holstein cultures continued to maintain elevated expression of TCF7, Angus cultures had started to return to basal levels of expression at 8 h post LPS, although expression was still significantly higher (*P* < 0.05) than without treatment.

Similar to old cultures, Holstein cultures had significantly (*P* < 0.05) higher expression of FES at 0 and 2 h post LPS treatment, with expression levels being 3.7 and 5.4-fold higher, respectively, and a trend (4.8-fold, *P* < 0.10) towards higher expression in Holstein cultures at 8 h post LPS. PIK3R1 also had higher levels of expression (*P* < 0.01) 2 h post-LPS treatment in Holstein cultures. This difference was due to a significant (*P* < 0.01) 4-fold decrease in PIK3R1 gene expression in Angus cultures that was not detected in Holstein cultures. By 8 h post LPS treatment, PIK3R1 expression returned to near-basal levels in Angus cultures.

Most noteworthy was the gene, *NFATC1*, which showed a very similar gene expression pattern between breeds as was seen in the age difference. Both Angus and Holstein cultures significantly increased their expression of NFATc1 at 2 h post LPS treatment (30-fold and 12-fold respectively), with Holstein cultures having a 6.5-fold higher (*P* < 0.01) level of expression over Angus cultures at 2 h post LPS. Holstein cultures also trended towards greater expression (2.2 and 1.7-fold, *P* < 0.10) at 0 and 8 h post-LPS. By 8 h post LPS, both breeds returned to basal levels of expression. Finally, RORA, had higher (*P* < 0.01) levels of expression in Holstein cultures at 0 and 2 h post LPS treatment. At 0 h, Holstein cultures had a 2.2-fold higher level of expression and at 2 h they had a 2.0-fold higher level of expression. No LPS treatment effect was detected.

## Discussion

An increase in the systemic production of pro-inflammatory mediators has been well characterized as a component of the aging process, heightening the risk of cardiovascular, neurodegenerative and other diseases in humans. This process, coined by Franceschi et al.,[34] as “inflamm-aging”, is highlighted by increased concentrations of serum TNF-α, IL1-ß and IL-6 along with acute phase proteins, such as C reactive protein [34, 35]. To determine the potential mechanism behind an increase in chronic inflammation associated with age, both in healthy aging individuals and during the diseased state, regulation of pro-inflammatory gene expression by DNA methylation has been widely studied.

A substantial decrease in methylation at a single CpG site in the promoter of the *IL6* gene was measured in patients with rheumatoid arthritis versus healthy controls. In the same study, ranking of healthy control macrophages in response to LPS showed that an increase in LPS-induced IL-6 production was correlated with a decrease in *IL6* promoter methylation [36]. Epigenetic regulation of another pleiotropic pro-inflammatory cytokine gene, *TNF,* has been similarly described. In comparison to healthy controls, patients with previous exposure to the Dengue virus have higher blood TNF-α expression with a concomitant decrease in promoter methylation [37]. DNA methylation changes have also been reported to occur in *TNF* in humans as part of the natural aging process. Macrophages obtained from 78 healthy individuals showed a 1.4% per decade decrease in three 5’ CpG motifs located with the *TNF* promoter [38]. Increased methylation of *TLR4*, the key TLR in response to LPS, has also been shown in the context of intestinal epithelial homeostasis, whereby intestinal epithelial cells are made tolerant to stimulation by commensal microbiota through down-regulation of TLR4 to avoid continuous inflammation [39]. In that study, a comparison between a high responding and a low responding intestinal epithelial cell line showed that the lower responding cells not only exhibit decreased expression but also enhanced methylation in 11 CpG sites in the gene promoter of the *TLR4* gene. Furthermore, inhibition of DNA methylation with AZA treatment in these two cell lines abolished the difference in TLR4 expression, further establishing a causal role for DNA methylation in the regulation of TLR4 [39].

Research on epigenetic regulation of the immune response in livestock species has been somewhat more limited. However, it has been shown that in response to LPS treatment, bovine PBMCs decrease expression of methylation and histone deacetylation enzymes, suggesting LPS treatment may result in lower gene methylation and increased histone acetylation [40]. The effects of *Mycobacterium bovis* infection on global DNA methylation and gene expression have also been reported in bovine CD4+ T- cells, whereby, DNA methylation was negatively correlated with expression of interferon-γ (*IFNG*) and a gene that encodes the OX-40 receptor (*TNFRSF4*). The two genes, *IFNG* and *TNFRSF4*, are involved in T-cell response and proliferation, respectively, and provide one potential mechanism that contributes to the shift from Th1 to Th2 T-cell response following bovine tuberculosis infection [41]. Epigenetic mechanisms have also been shown to play a role in the hepatic expression of TLR4, lipopolysaccharide binding protein (LBP) and haptoglobin in response to *E. coli* mastitis. Chromatin de-compaction and increased expression of key innate response genes, *LBP, HP, TLR2* and *TLR4* occurs in liver biopsies following an intra-mammary challenge with *E. coli*. In *TLR4,* the chromatin changes are also correlated with promoter de-methylation [42]. In a similar study that assessed the effects of feeding a high concentrate diet to induce sub-acute ruminal acidosis (SARA), chromatin de-compaction and expression of these innate response genes was again increased in liver biopsies with subsequent DNA de-methylation seen in all four genes. Presumably, these changes were a result of greater concentrations of LPS found in the rumen, and hepatic and portal veins of cows experiencing SARA [43].

To elucidate the underlying causes of differences in innate response to mastitis causing pathogens, we have taken advantage of two phenotypes with clear response differences, age and breed, where fibroblasts from older versus younger animals [22], and Holstein versus Angus animals [26] have much higher in vitro responses. The age-dependent difference in response has also been shown to be at least partially mediated by epigenetic mechanisms. Inhibition of DNA methylation and histone deacetylation in fibroblasts isolated from the same animal at 5- and 16-months of age abolishes differences in in vitro cytokine production suggesting methylation, chromatin modifications, or both contribute to the age-dependent increase in response [21]. To determine which genes are regulated by DNA methylation, methylated CpG island recovery assay or MIRA-seq, was previously performed on both the age and breed phenotypes following RNA-seq analysis. Unfortunately, this technique, which utilizes the protein complex MBD2b-MBD3L1 complex to enrich for methylated regions within the genome, has only moderate resolution (~500 bp) and few differences were detected in both phenotypes that fell within gene regions and none of these differences were detected in regions with a role in the innate response. Since it is known that small changes in methylation can be effective in changing gene expression it was necessary to utilize a more sensitive technique, such as was performed in the current study.

### Methylation differences in 5- versus 16-month dermal fibroblasts

Reduced representation bisulfite sequencing was performed on fibroblasts taken from 5- and 16-month old heifers that had significantly different IL-8 and IL-6 protein production and gene expression. This analysis revealed 14,094 differentially methylated CpG sites. Approximately two-thirds of these sites were more methylated in younger cultures, consistent with previous data suggesting global hypo-methylation in older individuals [44, 45]. The transcription factor, NF-κB which is a major activator of pro-inflammatory gene expression downstream of a number of pattern recognition receptors and TNF-associated receptors [46], was identified as significantly associated with hyper-methylated promoter regions in young cultures. This suggests that increased methylation in young cultures may block NF-κB binding sites, reducing transcription of pro-inflammatory genes. Blocking of NF- κB gene transcription by DNA methylation has been shown to occur in patients with secondary acute myeloid leukemia (sAML), where hematopoietic progenitor cells fail to express Fas receptor, an activator of apoptosis. The *FAS* gene was shown to contain 3 canonical NF-κB binding sites and increased methylation in bone marrow blast cells from patients with sAML as compared to those with low-risk myelodysplastic syndrome whose Fas expression remains intact. In addition, re-expression of Fas occurred in sAML patients upon de-methylation with AZA [47]. Although only identified in our suggestive (≥5× coverage) analysis, the transcription factor, cAMP regulated binding protein (CREB), was also associated with gene promoters more methylated in young cultures. Downstream of a wide variety of serine-threonine kinases (such as PKA, PKC, and p38 MAPK) CREB regulates a multitude of processes, including the innate immune response [48]. Recently, CREB has been shown to mediate TNF-α dependent GM-CSF production in primary asthmatic lung fibroblasts and human fetal lung fibroblasts [49]. CREB also increases LPS and TLR4 dependent IL-6 production in vascular smooth muscle cells which may contribute to the vascular inflammation seen in atherosclerosis [50].

In older cultures, transcription factor binding sites for Sp1, p300 and ATF, were enriched in hyper-methylated genes. Numerous genes contain Sp1 binding sites, which are located in GC-rich regions often regulated by DNA methylation. Somewhat contradictory to our hypothesis, Sp1 does up-regulate expression of some innate response genes. In intestinal epithelial cells, basal expression of TLR5 is regulated by Sp1. Interestingly, inducible expression of TLR5 is also partially mediated by p300, in agreement with our results that both Sp1 and p300 regulated genes were hyper-methylated in older cultures [51]. Alternatively, in response to LPS, Sp1 enhances macrophage transcription of IL-10, a key anti-inflammatory cytokine [52]. ATFs are members of the activating transcription factor/cAMP responsive element binding protein (ATF/CREB) family of transcription factors. It was not clear with DAVID analysis which of the family members were enriched in regulation of the genes more methylated in older cultures, however, analysis of just the 106 genes regulated by ATF showed that 62 were regulated by ATF6. ATF6 is a key transcription factor that is activated under ER stress and mediates the unfolded protein response. Again, contrary to our data, ER stress contributes to inflammation during an infection with *Brucella abortis* in a NOD1/NOD2 dependent manner and the unfolded protein response has been shown to strengthen NF-κB dependent inflammation [53, 54]. However, other ATFs, such as ATF3 have been shown to have the opposite effect and reduce inflammatory gene expression [55]. Furthermore, when additional stringency was applied to the TFBS analysis to include only genes that contained differentially methylated sites with ≥10× coverage we failed to identify any transcription factors associated with genes hyper-methylated in old cultures, unlike what was found in young cultures. An additional caveat to the transcription factor binding data used in the current study is that conclusions were drawn on sequence conservation found between human and rodent alignments. Sequence conservation does not necessarily result in transcription factor binding and regulation and whether enrichment of the identified transcription factors regulate cellular response differences to LPS in a methylation-dependent manner would need to be validated in our model.

### Gene expression analysis of differentially methylated genes

Methylation of DNA is most known for its role in repressing gene transcription. To determine if the measured methylation differences had any effect on gene expression, we chose six genes that exhibited higher methylation in young cultures for RT-qPCR analysis. We hypothesized these six genes would have lower expression in the young cultures due to their increased methylation status. We chose the following six genes for their potential role in the innate response: *TNFSF13, PIK3R1, NFATC1, FES, TCF7* and *RORA*. The TNF superfamily 13, or APRIL, protein is a surface or secreted ligand recognized by the transmembrane activator and calcium-modulating cyclophilin ligand interactor (TACI) and B-cell maturation antigen (BCMA) receptors and has been implicated in the pathogenesis of numerous pro-inflammatory conditions such as rheumatoid arthritis [56], atherosclerosis [57], lupus [58] and psioriasis [59]. Moreover, APRIL has been shown to activate NF-κB dependent cytokine production in macrophages [60] and keratinocytes [59]. In our analysis, fibroblast expression was significantly up-regulated in older cultures at 0 and 8 h post LPS treatment as we expected. The single site hyper-methylated in young cultures was located near to the consensus NF- κB binding sequence, 5′-GGGRNYY YCC-3′. However, since the gene did not increase after LPS treatment and was already up-regulated under basal conditions, it is unlikely NF-κB regulates its transcription in response to LPS.

The gene, *PIK3R1*, transcribes the p85α subunit of phosphoinositide 3-kinase (PI3K), a lipid kinase that phosphorylates phosphatidylinositol 4,5-bisphosphate (PIP2) to phosphatidylinositol 3,4,5-trisphosphate (PIP3). The p85α subunit is a regulator of kinase activity, whereby it is thought to inhibit PI3K activity under most circumstances [61]. Interestingly, in a murine model of polymicrobial sepsis, inhibition of the PI3K pathway increased serum IL1-β, IL-6 and TNF-α levels and decreased the survival rate of septic mice [62]. Alternatively, pharmacological activators of PI3K signaling have recently been suggested as anti-inflammatory drug candidates [63]. However, the role of PI3K in inflammation is complex, since kinase activity can also activate leukocytes and is further complicated by the existence of multiple isoforms of PI3K with a variety of physiological functions [64]. In our model, PIK3R1 expression was up-regulated in older cultures at all 3 time points post LPS with a fairly consistent fold change of 2.5 – 3.0 fold. A single CpG site in the promoter region was more highly methylated, only 23 base pairs from start site of the gene, potentially interfering with the progression of the RNA polymerase enzyme complex.

Nuclear factor of activated T-cells, calcineurin dependent 1 (NFATc1), the gene product of *NFATC1,* is a calcium dependent transcription factor that is necessary for lymphocyte development and is expressed in a number of other cell types such as dendritic cells [65], macrophages [66] and endothelial cells [67] and is responsible for mediating TLR-4 independent, CD-14 dependent pro-inflammatory gene expression in response to microbial agonists, such as LPS. Interestingly, the *NFATC1* gene is less methylated and more expressed in individuals with lower socioeconomic status which is a known risk factor for increased inflammation that can lead to pathologies such as type II diabetes [68, 69]. In our study, NFATc1 was induced following LPS stimulation in both young and old fibroblasts, further solidifying its role as an LPS-responsive transcription factor. In the promoter, the differentially methylated CpG was located directly downstream of a Sp1 binding site that has been shown in numerous recent studies to bind at lower levels with increased DNA methylation status [70–72]. In addition, two other sites were located in the last intron of the gene. This is of interest because an enhancer element has been identified using human lymphocytes within the last intron of the NFATc1 gene that activates transcription to an even greater extent than the promoter region of the gene [73].

Feline sarcoma oncogene (FES) is a non-receptor tyrosine kinase that, in humans, stimulates hematopoiesis, osteoclastogenesis and mast cell activation [74]. It is worth mentioning that both FES and PI3-K activate mast cells through a similar SCF-c-Kit-integrin pathway and the two proteins have been speculated to interact with one another through tyrosine phosphorylation of PI3-K by FES [75, 76]. As with PIK3R1, FES kinase expression was also similarly up-regulated at all time points in older cultures while younger cultures had two hyper-methylated sites near to the transcriptional start site which again may interfere with transcriptional elongation by RNA polymerase.

Transcription factor 7 (TCF7), transcribed from the gene *TCF7* is a transcription factor that is crucial to the proper development of T-cells in the thymus, but has been shown to inhibit the development of regulatory T-cells, or Tregs, which suppress inflammation and aid in the resolution of infection [77]. Although several methylation differences were detected within the promoter region of *TCF7*, no difference in expression was measured in fibroblasts either basally or post LPS. There are a couple of possible explanations for this discrepancy. Methylation itself may not be sufficient to repress expression and it is likely an over-simplification to assume methylation always leads to repression. For example, in a study assessing the relationship between methylation and gene transcription in the blood of 148 healthy human subjects, the authors found that 276 of 798 local associations were actually positively associated, where less methylation led to less transcription or more methylation led to more transcription [78]. Other factors, such as histone modifications and transcription factors that also have an effect on transcription should be taken into consideration as they can override any differences in methylation as has been shown in the differentially imprinted gene *IGF2R* [79]. Lastly, it is possible that splice variants of *TCF7* exist that exhibit differential expression in our samples but were not measured in our RT-qPCR analysis.

Finally, RORA, or retinoic acid related (RAR) orphan receptor alpha, belongs to a family of nuclear receptor transcription factors (RORs) which have known roles in the innate and adaptive immune response, circadian rhythm and metabolic regulation, among others [80]. Specifically, RORA is required for the development of type II innate lymphoid cells and mice lacking the gene experience reduced eosinophil-mediated lung inflammation in response to allergens [81] and are less susceptible to obesity induced inflammation in response to a high fat diet [82]. We detected a small, but significant increase in RORA expression in older fibroblasts at 8-h post LPS. In this case, we identified 6 methylation differences all within first intron of the gene which can contain enhancer elements for gene activation [83].

### Expression of differentially methylated genes in Angus versus Holstein fibroblasts

In our final experiment, we wanted to determine whether similar expression differences could be detected in another high-low response phenotype, a breed difference identified in our lab that shows the Holstein breed having a much higher in vitro response as compared to the Angus breed [26]. Genes similarly up-regulated in Holsteins might suggest a conserved mechanism of regulation, such as DNA methylation or other epigenetic modifications. This of particular interest in the breed difference as these two breeds are not only genetically selected for divergent purposes but also experience highly dissimilar neonatal environments, leaving room for possible environmental interventions. Many studies have shown that neonatal nutrition, maternal care and exposure to immune agonists can shape an individual’s adult epi-genotype and all three of these environmental influences differ between Holstein and Angus calves. Angus calves are maternally cared for in a pasture setting, drink milk from their dam, and are exposed to a variety maternally-derived commensal and environmental microbes in contrast to Holstein calves that are raised individually without maternal contact, fed milk replacer, and are housed in a considerably more defined environment.

Expression analysis of fibroblasts isolated from the two breeds showed some similar differences to our age-dependent differences. In the high responding Holstein breed, *FES, NFATC1, RORA,* and to a lesser extent *PIK3R1,* were up-regulated. Additionally, *TCF7* was up-regulated, a difference not detected in the young-old culture comparison. This discrepancy may indicate a genetic difference as a result of selection for different traits (beef versus dairy) between Angus and Holstein breeds. No difference was measured in *TNFSF13* suggesting the loss of methylation is unique to the aging process. Most noteworthy of the differentially expressed genes were *FES* and *NFATC1*. These two genes showed an identical pattern of up-regulation in Holstein cells as was seen in older cells, indicating they may be similarly regulated. However, additional experiments with the Holstein and Angus cultures would be necessary to confirm whether expression differences are due to differences in methylation or other epigenetic factors, genetic differences, or a combination of factors. Expression of *FES* is up-regulated basally in both high response phenotypes, making it a candidate biomarker for selection strategies which would require a rapid test without the need of culturing cells and treating with LPS. Additionally, *NFATC1* is of interest due to its role in the response to LPS and as a known contributor to many pro-inflammatory pathologies. Future studies assessing methylation differences in Angus and Holstein breeds are underway and should provide insight into how this phenotype is regulated.

One limitation of the current study was that the RRBS technique does not allow for a comprehensive methylation analysis and as a result many CpG sites are missed. This may be one reason why we failed to identify methylation differences in classic LPS response genes that show similar up-regulation of expression in both high responding phenotypes. Targeted bisulfite sequencing of candidate genes, such as *TLR4* and *IL8*, will be the subject of future experiments to determine whether methylation may influence expression of genes that directly mediate LPS response.

## Conclusions

Overall, the current study has shown that many sites differ in their methylation status in young versus old cultures, with the majority of sites becoming less methylated as the animals increased in age. Candidate innate response genes that were hyper-methylated in young cultures also exhibited lower expression in most instances, indicating methylation may suppress expression in younger cultures making them less responsive to TLR agonists, such as LPS. Expression analysis in a second high-low response phenotype, Angus versus Holstein fibroblasts, identified two genes (*FES* and *NFATC1*) that showed an identical response pattern as the young and old cultures. These two genes may be useful as candidate biomarkers for predicting whether an animal will have a high or low response phenotype, and subsequently, whether she is susceptible to severe mastitis. In conclusion, knowledge of critical changes in methylation that increases the magnitude of inflammatory response within an individual may elude to the mechanisms of between-individual variation in the innate response. This is particularly important in diseases where an unregulated innate response contributes to the pathology of the disease, such is the case in bovine mastitis.

## Additional files

Additional file 1: Hyper-methylated genes in 5-month cultures. **Table S1.** Hyper-methylated promoter regions. **Table S2.** Hyper-methylated exon regions. **Table S3.** Hyper-methylated intron regions. (XLSX 209 kb)

Additional file 2: Hyper-methylated genes in 16-month cultures. **Table S1.** Hyper-methylated promoter regions. **Table S2.** Hyper-methylated exon regions. **Table S3.** Hyper-methylated intron regions. (XLSX 129 kb)

Additional file 3: Clustering analysis on the top 100 differentially methylated sites in young (1A - 6A) versus old cultures (1B - 6B). (PDF 276 kb)

## Abbreviations

ATF: Activating transcription factor
AZA-TSA: 5-aza-2-deoxycytidine and Trichostatin A
CREB: cAMP regulated binding protein
DAVID: Database for Annotation, Visualization, and Integrated Discovery
DMC: Differentially methylated CpG
DMR: Differentially methylated region
*E. coli*: *Escherichia coli*
FES: Feline sarcoma oncogene
GO: Gene Ontology
IL-6: Interleukin-6
IL-8: Interleukin-8
KEGG: Kyoto Encyclopedia for Genes and Genomes
LPS: Lipopolysaccharide
MIRA: Methylated CpG island recovery assay
Mr: Methylation ratio
NFATc1: Nuclear factor of activated T-cells, calcineurin dependent 1
PAMP: Pathogen associated molecular pattern
PI3K: Phosphoinositide 3-kinase
PIK3R1: Phosphatidylinositol 3-kinase regulatory subunit alpha
PRR: Pattern recognition receptor
RORA: Retinoic acid related orphan receptor alpha
*S. aureus*: *Staphylococcus aureus*
sAML: Secondary acute myeloid leukemia
SARA: Sub-acute ruminal acidosis
TCF7: Transcription factor 7
TFBS: Transcription factor binding site
TLR: Toll-like receptor
TNFSF13: Tumor Necrosis Factor Super Family 13
TSS: Transcription start site
WGBS: Whole genome bisulfite sequencing
