# Supplementary figures and images for: Genome-wide methylation analysis reveals differentially methylated loci that are associated with an age-dependent increase in bovine fibroblast response to LPS
Source: BMC Genomics. 2017 May 25;18:405. doi: 10.1186/s12864-017-3796-1 (PMC5445414; doi:10.1186/s12864-017-3796-1)

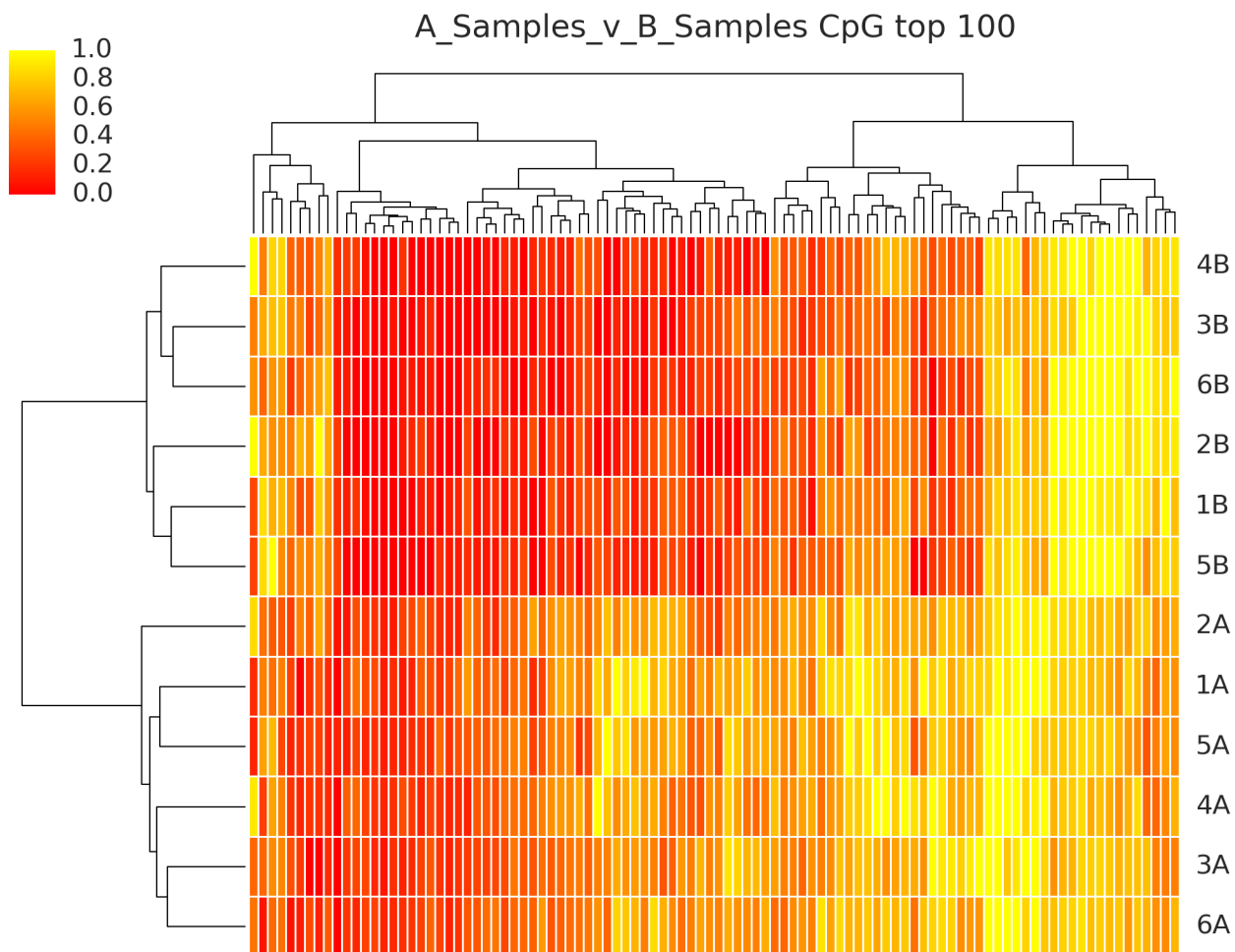

Supplement: Supplementary file 3 — Clustering analysis on the top 100 differentially methylated sites in young (1A - 6A) versus old cultures (1B - 6B). (PDF 276 kb) [file 12864_2017_3796_MOESM3_ESM.pdf]
